# Candidate Porcine *Kobuvirus,* China

**DOI:** 10.3201/eid1505.081518

**Published:** 2009-05

**Authors:** Jie-mei Yu, Miao Jin, Qing Zhang, Hui-ying Li, Dan-di Li, Zi-qian Xu, Jin-song Li, Shu-xian Cui, Su-hua Yang, Na Liu, Zhao-jun Duan

**Affiliations:** China Center for Disease Control, Beijing, People’s Republic of China

**Keywords:** Viruses, kobuvirus, reverse transcription–PCR, China, letter

**To the Editor**: The picornaviruses constitute a large, diverse family of positive-sense RNA viruses, which comprise 8 genera: *Enterovirus*, *Aphthovirus*, *Cardiovirus*, *Hepatovirus*, *Parechovirus*, *Erbovirus*, *Kobuvirus*, and *Teschovirus* (*1*). The genus *Kobuvirus* contains 2 known species: *Aichi virus,* which was identified in humans in 1989 and was found to be associated with human acute gastroenteritis (*2*), and *Bovine kobuvirus,* which was identified in 2003 in apparently healthy cattle (*3*). In our study, we identified a candidate novel strain of kobuvirus from porcine fecal specimens; this strain is markedly different from Aichi virus and bovine kobuvirus.

Using reverse transcription–PCR (RT-PCR) to characterize calicivirus in porcine fecal specimens with a primer pair of p289/VN3T20 designed for a 3-kb fragment of the virus, we observed an unexpected band on agarose gel electrophoresis (*4*). After purification and sequencing, the 1,185-bp fragment was found to share 73% similarity with the 3D region of bovine kobuvirus. A pair of primers was then designed from this sequence and synthesized (forward: 5′-TGGACGACCAGCTCTTCCTTAAACAC-3′ and reverse: 5′-AGTGCAAGTCTGGGTTGCAGCCAACA-3′; 495 bp) to screen other porcine samples for the virus by PCR. Our samples were 322 fecal specimens collected during 2006–2007 from healthy piglets <15 days of age from 3 different farms and several sporadically distributed families that raised pigs in Lulong County, China. Of the 322 samples, 97 were positive. All products were sequenced, and 18 were chosen randomly for deposit in GenBank under accession nos. FJ459895–FJ459912.

To further characterize the virus, we designed primers corresponding to the viral protein (VP) 0 region of kobuvirus on the basis of conserved sequences deduced by comparing the sequences of bovine kobuvirus (GenBank accession no. NC_004421) and Aichi virus (GenBank accession no. NC_001918). An 823-bp fragment was examined and then submitted, together with the 1185-bp sequence, to GenBank under accession no. FJ493623. The obtained sequences were analyzed by using the DNASTAR software package (www.dnastar.com) and were compared with other sequences in GenBank by using BLAST (www.ncbi.nlm.nih.gov/blast/Blast.cgi).

The results demonstrate that the 3D partial region of the novel strain has nucleotide homology of 73% and 70% to that of bovine kobuvirus and Aichi virus, respectively. The sequence in the VP0 region was less conserved; nucleotide and amino acid homologies were 69% and 71%, respectively, to those of bovine kobuvirus and 66% and 69%, respectively, to those of Aichi virus.

Partial sequences of the 3D region have been used to deduce phylogenetic and taxonomic relationships among picornaviruses; these sequences have been particularly useful for placing viruses within species or genera or for comparing viruses of different genera or families (*5*). We constructed phylogenetic trees by using MEGA software version 3.1 (www.megasoftware.net). The phylogenetic analysis showed a single genetic lineage for the novel virus, close to *Kobuvirus* but phylogenetically distinct from both bovine kobuvirus and Aichi virus, which suggests that the porcine fecal specimen contained a candidate novel species of *Kobuvirus* (Figure).

**Figure:**
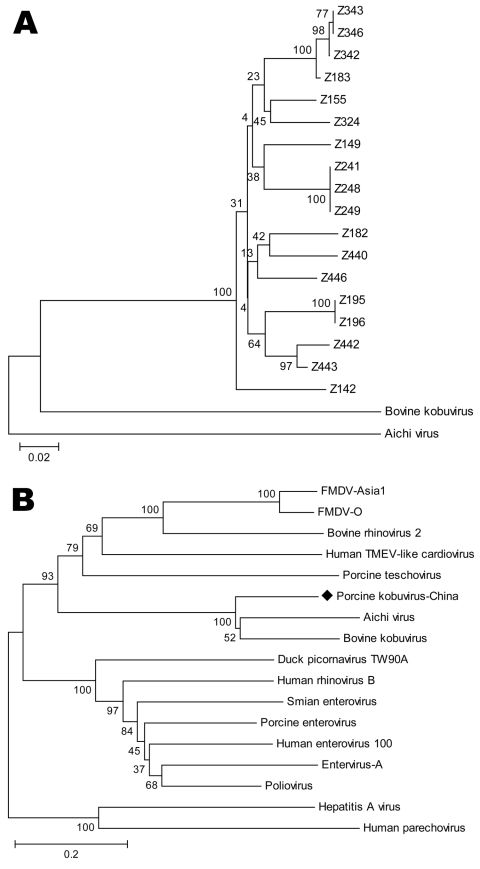
A) Phylogenetic tree of the partial sequences in the 3D region of the candidate novel virus, Aichi virus, and bovine kobuvirus. B) Relationships between the candidate novel virus and other picornaviruses based on nucleotide differences in the 3D region. FMDV, foot and mouth disease virus; TMEV**,** Theiler's murine encephalomyelitis virus. Scale bars indicate nucleotide substitutions per site.

The filtered fecal samples positive for the virus were inoculated onto RD cells. After 3 serial passages, no obvious cytopathic effect in RD cells was noted. The cells and supernatants in every passage were collected separately for RNA extraction. We then used semiquantitative RT-PCR with glyceraldehyde-3-phosphate dehydrogenase as an internal reference to detect the virus and determine the amount of the viral RNA in the medium. Cells and supernatants of the 3 passages were virus positive, and the control inoculated with phosphate-buffered saline was virus negative. However, with each passage, the amount of viral RNA in the medium decreased. Whether the positive result was caused by residual viruses of the initial inoculation or by the decreased propagation of the virus in the cells is not clear. Further studies, such as continuous serial passages and neutralization assay, are needed to determine the final activity of the virus in RD cells, as well as in other cells such as Vero and HeLa, because several species of picornaviruses have been identified as causing persistent infections in these cells in vitro (*6*–*10*).

In conclusion, we report the genetic characterization and biological properties of a new agent in China. Of note, while we were preparing this article, a similar article from Hungary was published (*5*). After comparing our 1,185-bp sequence with the sequence from Hungary, we found that our sequence was 171 bp longer at the 3′ end and 50 bp shorter at the 5′ end and that the truncated sequence in the middle (same length) had a nucleotide homology of 92.1%. Phylogenetic analysis indicated that the 2 sequences may share the same origin (Figure, panel B). In addition, prevalence of our virus (30.12%) was higher than that of the virus from Hungary (13.3%). Further studies are needed to determine the complete genome and the relevance of the candidate porcine *Kobuvirus* as a causative agent of disease in pigs and a potential zoonotic agent.
